# Light helicity detector based on 2D magnetic semiconductor CrI_3_

**DOI:** 10.1038/s41467-021-27218-3

**Published:** 2021-11-25

**Authors:** Xing Cheng, Zhixuan Cheng, Cong Wang, Minglai Li, Pingfan Gu, Shiqi Yang, Yanping Li, Kenji Watanabe, Takashi Taniguchi, Wei Ji, Lun Dai

**Affiliations:** 1 State Key Lab for Artificial Microstructure & Mesoscopic Physics and Frontiers Science Center for Nano-optoelectronics, School of Physics, Peking University, Beijing, 100871 China; 2 Collaborative Innovation Center of Quantum Matter, Beijing, 100871 China; 3 Beijing Key Laboratory of Optoelectronic Functional Materials & Micro-Nano Devices, Department of Physics, Renmin University of China, Beijing, 100872 China; 4 Academy for Advanced Interdisciplinary Studies, Peking University, Beijing, 100871 China; 5 National Institute for Materials Science, 1-1 Namiki, Tsukuba, 305-0044 Japan; 6 Peking University Yangtze Delta Institute of Optoelectronics, Beijing, 100871 China

**Keywords:** Magnetic devices, Magnetic properties and materials, Two-dimensional materials

## Abstract

Two-dimensional magnetic semiconductors provide a platform for studying physical phenomena at atomically thin limit, and promise magneto-optoelectronic devices application. Here, we report light helicity detectors based on graphene-CrI_3_-graphene vdW heterostructures. We investigate the circularly polarized light excited current and reflective magnetic circular dichroism (RMCD) under various magnetic fields in both monolayer and multilayer CrI_3_ devices. The devices exhibit clear helicity-selective photoresponse behavior determined by the magnetic state of CrI_3_. We also find abnormal negative photocurrents at higher bias in both monolayer and multilayer CrI_3_. A possible explanation is proposed for this phenomenon. Our work reveals the interplay between magnetic and optoelectronic properties in CrI_3_ and paves the way to developing spin-optoelectronic devices.

## Abstract (web summary)

Two-dimensional magnetic semiconductors hold promise for spin- and valleytronic applications. Here, the authors report the realization of light helicity detectors based on graphene/CrI_3_ van der Waals heterostructures, exhibiting a photocurrent behaviour determined by the magnetic state of CrI_3_.

## Introduction

Since the first discovery of intrinsic ferromagnetism in two-dimensional (2D) van der Waals (vdW) crystals of Cr_2_Ge_2_Te_6_ and CrI_3_ in 2017^1,2^, 2D magnetic materials have aroused wide research interest. They provide a platform for studying light–matter interactions and magneto-optical/electrical phenomena at the atomically thin limit and also promise magneto-optoelectronic devices application^3^. Monolayer CrI_3_ has ferromagnetic (FM) nature, whereas multilayer CrI_3_ has a layered antiferromagnetic (AFM) nature with an easy magnetization axis perpendicular to the layers^2,4–6^. To date, various studies about CrI_3_ were reported, including helical luminescence^7^, tunneling magnetoresistance^5,6,8^ and electrostatic doping^9^, which demonstrated that the optical/electrical properties of CrI_3_ are coupled with its magnetic property. To further study 2D magnetic semiconductor materials and advance their application, understanding the interplay between their magnetic and optoelectronic properties and developing magneto-optoelectronic devices are indispensable.

In this work, we fabricate light helicity detectors based on graphene-CrI_3_-graphene vdW heterostructures and study their helical photoresponse properties via magneto-optoelectronic measurements. Devices with two representative thicknesses (monolayer and multilayer) of CrI_3_ are investigated. We investigate their circularly polarized light excited current and reflective magnetic circular dichroism (RMCD) under various magnetic fields *μ*_0_*H* and temperatures. The devices exhibit helicity-selective photoresponse behavior determined by the magnetic state of CrI_3_. Both the helicity-dependent photocurrent and illumination-dependent tunneling current are studied in detail. The calculated photoresponsivity polarization has a clear relation with magnetic field, consistent with the RMCD signals. For the monolayer CrI_3_ device, when the applied magnetic field switches the CrI_3_ between the up magnetized state (*μ*_0_*H* > 0.1 T) and the down magnetized state (*μ*_0_*H* < −0.1 T), the photoresponsivity polarization ($\rho$) changes between ~−6 and +6%, and the RMCD signal changes between ~+1 and −1%. The photoresponsivity polarization equals zero, i.e., the helicity-dependent photoresponse phenomenon vanishes, at temperature higher than 40 K, close to the Curie temperature (*T*_c_) of monolayer CrI_3_ (45 K)^2^. For the multilayer (14 nm thick) CrI_3_ device, $\rho$ value displays multiple plateaus with magnetic field sweeping, and saturates at ~±4.5% when | *μ*_0_*H* | > 2.2 T, corresponding to different spin configurations in CrI_3_ enabled by spin-flip transitions at different magnetic fields. Specifically, for both monolayer and multilayer CrI_3_ devices, the light-on current decreases with increasing excitation power at a higher bias. Based on detailed discussions, we provide a possible explanation to this phenomenon. Our work reveals the interplay between magnetic and optoelectronic properties in CrI_3_, and paves the way to developing spin-optoelectronic devices.

## Results

### Device fabrication and characterization

**Fig. 1:**
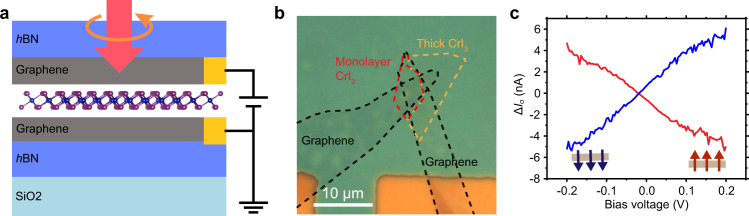
The monolayer CrI_3_ light helicity detector D1. Schematic diagram (**a**) and optical micrograph (**b**) of a monolayer CrI_3_ (demarcated by the red dashed lines) light helicity detector D1 with few-layer graphene contacts (demarcated by the black dashed lines). The yellow dashed lines demarcate the thick CrI_3_, which was not illuminated by the excitation light in the measurements. The device is encapsulated by *h*BN flakes. **c** The difference of the light-on currents (Δ*I*_σ_ = *I*_+_ − *I*_−_) under ${\sigma }^{+}$ and ${\sigma }^{-}$ excitations as function of the bias, measured at *μ*_0_*H* = 0 T. Before being measured, the monolayer CrI_3_ device was fully magnetized at | *μ*_0_*H* | > 0.3 T. The red (blue) color denotes up (down) magnetized CrI_3_. The Δ*I*_σ_–*V* curve shows negative (positive) slope for up (down) magnetized state of monolayer CrI_3_. These results show that the photocurrents in a fully magnetized CrI_3_ is helicity-dependent.

The CrI_3_ based light helicity detector was fabricated by mechanically exfoliating and dry transferring few-layer graphene, CrI_3_, and hexagonal boron nitride (*h*BN) flakes from their bulk crystals to construct graphene-CrI_3_-graphene heterostructure. To avoid degradation^8^, the device was encapsulated by *h*BN flakes. The top and bottom graphene layers act as the electrodes. All the procedures were conducted in a glove box within an inert atmosphere (<0.1 ppm of water and oxygen) (see Methods). Figure 1a, b show the schematic diagram and optical image, respectively, of a monolayer CrI_3_ light helicity detector D1. We measured the circularly polarized light excited current and RMCD of the device under various magnetic fields and temperatures. Both the excitation light and magnetic field *μ*_0_*H* are perpendicular to the 2D layers and parallel to the easy magnetization axis of CrI_3_ (see Methods). Notably, in the experiment, the beam diameter of the excitation light is about 1 μm, less than the scale of vertical tunneling junction. For photoresponse and RMCD measurements, the temperature is 2 K and the excitation power is 10 μW, unless otherwise specified.

### Circularly excited light-on currents

The monolayer CrI_3_ device was first fully magnetized at |*μ*_0_*H*| > 0.3 T. Then its light-on currents (*I*_light_) were measured at *μ*_0_*H* = 0 T under both right circularly polarized light (${\sigma }^{+}$) and left one (${\sigma }^{-}$) excitations. Their difference (Δ*I*_σ_ = *I*_+_ − *I*_−_) is plotted as a function of applied bias (*V*) in Fig. 1c, where ${I}_{\pm }$ are the light-on currents under ${\sigma }^{\pm }$ excitations. The Δ*I*_σ_–*V* curves exhibit distinguishable difference between the *I*_light_ under ${\sigma }^{+}$ and ${\sigma }^{-}$ excitations, with negative (positive) slope for the up (down) magnetized state of monolayer CrI_3_. These results show that the photocurrent in a fully magnetized CrI_3_ is helicity-dependent.

### Light Power dependent magneto-optoelectronic response

**Fig. 2:**
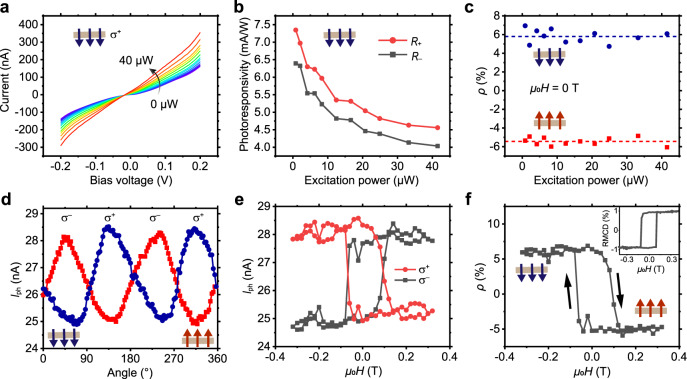
The magneto-optoelectronic response in the monolayer CrI_3_ device D1. **a** *I*–*V* curves of the device with a down magnetized state under ${\sigma }^{+}$ excitation with various powers (from 0 to 40 μW) measured at *μ*_0_*H* = 0 T (increased from −0.3 T). For identical bias, the light-on current (*I*_light_) increases monotonically with the excitation power. **b** Excitation power-dependent photoresponsivities (*R*_+_ and *R*_−_) measured at *V* = 0.2 V under ${\sigma }^{+}$ (red) and ${\sigma }^{-}$ (black) excitations in the down magnetized state CrI_3_. **c** Excitation power dependent photoresponsivity polarization $\rho$ measured at *μ*_0_*H* = 0 T. The $\rho$ is independent of excitation power with average values of ~−6% (red dashed line) for the up state (red dots) and ~+6% (blue dashed line) for the down state (blue dots). **d** Photocurrents, measured at *V* = 0.15 V, versus the angle of the quarter-wave plate. The up (down) state CrI_3_ exhibits a higher ${\sigma }^{-}$ (${\sigma }^{+}$) photocurrent. **e** Circularly excited photocurrents under various magnetic fields. The red (black) color denotes the ${\sigma }^{+}$ (${\sigma }^{-}$) excitation result. When *μ*_0_*H* sweeps between ±0.3 T, the photocurrent behaviors are opposite for ${\sigma }^{+}$ and ${\sigma }^{-}$ excitation. **f** The photoresponsivity polarization $\rho$ values and the reflective magnetic circular dichroism (RMCD) signals (the inset) at various *μ*_0_*H*. When *μ*_0_*H* sweeps between −0.3 and +0.3 T, $\rho$ changes between 6 and −6%, and RMCD signal changes between −1 and +1%. The CrI_3_ spin-flip transitions occur at ±0.1 T.

The excitation power dependent current versus bias relations (*I*–*V* curves) of a monolayer CrI_3_ with down magnetized state under ${\sigma }^{+}$ excitation at *μ*_0_*H* = 0 T (increased from –0.3 T) are shown in Fig. 2a. For identical bias, the *I*_light_ increases monotonically with the excitation power. We calculate photoresponsivity $R=\frac{{I}_{{{{{{\rm{ph}}}}}}}}{P}$, where *I*_ph_ is the photocurrent defined as *I*_ph_ = *I*_light_ – *I*_dark_, *I*_dark_ is dark current (i.e., tunneling current in dark), and *P* is the excitation power. The excitation power dependent *R*_+_ (under ${\sigma }^{+}$excitation) and *R*_−_ (under ${\sigma }^{-}$ excitation) at *V* = 0.2 V are plotted in Fig. 2b. The clear difference between the *R*_+_ and *R*_−_ illustrates again that the photocurrent is helicity-dependent and the device can serve as a light helicity detector^10^. The key parameter for a helicity detector is the photoresponsivity polarization defined as $\rho =\frac{{R}_{+}-{R}_{-}}{{R}_{+}+{R}_{-}}$, where ${R}_{\pm }$ are the ${\sigma }^{\pm }$ photoresponsivities^11^. Based on the above results, we obtain $\rho \approx -6$% (6%) for the up (down) magnetized monolayer CrI_3_ at *μ*_0_*H* = 0 T, which, represented by red (blue) dots in Fig. 2c, is independent of excitation power.

### Magnetic order dependence of the helical photocurrents

In order to further investigate the relation between the helical photocurrent and the intrinsic magnetism of the monolayer CrI_3_, we directly measured the photocurrent *I*_ph_ (*V* = 0.15 V) of D1 under various magnetic fields *μ*_0_*H* by the lock-in technique. The excitation laser is modulated to switch between ${\sigma }^{+}$ and ${\sigma }^{-}$ by a quarter wave plate equipped with a motorized rotation stage (see Methods). The circularly polarized photocurrent versus the phase of the quarter-wave plate relations for upstate (red dots) and downstate CrI_3_ (blue dots) are opposite (Fig. 2d), with *I*_ph_(${\sigma }^{+}$) > *I*_ph_(${\sigma }^{-}$) for the down state CrI_3_, and vice versa. When *μ*_0_*H* sweeps between ±0.3 T, the photocurrent behaviors are opposite for ${\sigma }^{+}$ and ${\sigma }^{-}$ excitation as shown in Fig. 2e. By curve fitting the data as shown in Fig. 2d with a cosine function, we can obtain the corresponding photoresponsivity polarization $\rho$. When *μ*_0_*H* sweeps between −0.3 and 0.3 T, $\rho$ changes between 6 and −6% (Fig. 2f), correspondingly, the RMCD signal changes between −1 and +1% (the inset in Fig. 2f). The monolayer CrI_3_ spin-flip transitions occur at ±0.1 T. While the device exhibits significant magnetic field-dependent helical photocurrent property, the dark tunneling current *I*_dark_ is almost independent of the magnetic field as shown in Supplementary Fig. 1, because the resistances of the down- or up-magnetized CrI_3_ are equal. All the above results demonstrate that the magnetic field-dependent circularly polarized photocurrent can be used to detect the helicity of the incident light.

**Fig. 3:**
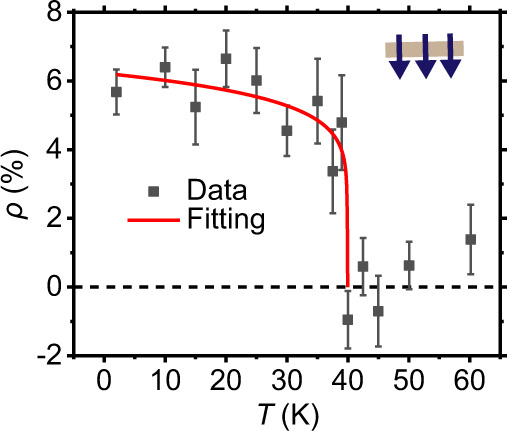
Temperature-dependent photoresponsivity polarization of the light helicity detector D1 measured at zero magnetic field. As temperature decreases from 80 to 2 K, $\rho$ (black dots) increases from 0% (the dashed line) to a saturated value of 6%, corresponding to the down magnetized state of CrI_3_. The helicities $\rho$, together with the error bars, are obtained by fitting the relation between the photocurrent and the angle of the quarter-wave plate. By fitting the data with $\rho \left(T\right)={\rho }_{0}{(1-\frac{T}{{T}_{{{{{{\rm{c}}}}}}}})}^{\beta }$ (red line), we obtain *T*_c_ = 40 K.

We also measured the photoresponsivity polarization $\rho$ of the helicity detector D1 under the zero-field-cooling condition (Fig. 3). When the temperature decreases from 80 to 2 K, $\rho$ increases from 0% to a saturation value of 6% (black dots). By curve fitting the data with $\rho \left(T\right)={\rho }_{0}{(1-\frac{T}{{T}_{c}})}^{\beta }$ (red line), where ${\rho }_{0}$ is the critical amplitude, $\beta$ is the critical exponent, we obtain *T*_c_ = 40 K, close to the Curie temperature of monolayer CrI_3_ (45 K).

The origin of the helicity-dependent photocurrent can be understood by analyzing the split energy band structure in a fully magnetized CrI_3_^12,13^ as shown in Supplementary Fig. 2a. The energy bands for electrons with different spins exhibit different band gaps. The narrower band gap corresponds to majority-spin electrons, and vice versa. Under the 633 nm light excitation, only the majority-spin electrons can be excited and form a few types of excitons^7,14^. These excitons obey the helicity-selective transition rule (Supplementary Fig. 2b), determined by the optical selection rules. From the frequency-dependent circularly polarized absorbance of ferromagnetic monolayer CrI_3_ at normal incidence^14^, we can see that, at the 633 nm (1.96 eV) excitation wavelength, ${\sigma }^{+}$ and ${\sigma }^{-}$ light absorbances are different, which are dominated by the B^+^ and B^−^ bright exciton absorbances, respectively, leading to the helicity-dependent photocurrent under applied bias. The helicity-dependent absorption is also demonstrated by the RMCD signals (the inset in Fig. 2f), which indicate the difference in reflectivity of the ${\sigma }^{+}$ and ${\sigma }^{-}$ light (see Methods).

### Helical photocurrents in multilayer CrI_3_ helicity detector

**Fig. 4:**
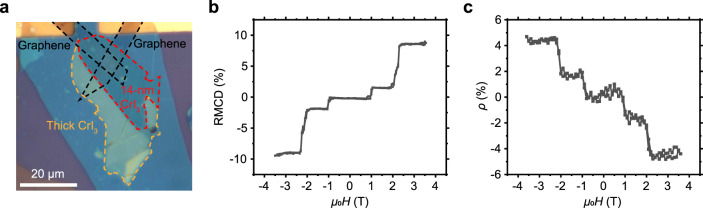
The multilayer CrI_3_ light helicity detector D2. **a** Optical image of the 14 nm thick CrI_3_ (demarcated by the red dashed lines) light helicity detector D2 with few-layer graphene contacts (demarcated by the black dashed lines). The yellow dashed lines demarcate the thick CrI_3_, which was not illuminated by the excitation light in the measurements. The device is encapsulated by *h*BN flakes. The reflective magnetic circular dichroism (RMCD) signals (**b**) and photoresponsivity polarization $\rho$ (**c**) versus *μ*_0_*H* relations of the device. Both RMCD and $\rho$ show five plateaus when *μ*_0_*H* sweeps between −3.5 and +3.5 T, and equal 0 at *μ*_0_*H* = 0 T, corresponding to the AFM ground state. RMCD signals and $\rho$ values saturate at ~±10 and ±4.5%, respectively, when | *μ*_0_*H* | > 2.2 T, corresponding to the fully spin-polarized states.

Figure 4a shows an optical image of a 14 nm thick CrI_3_ light helicity detector D2. The magnetoresistances measured in dark at *V* = 0.3 V under various magnetic fields are shown in Supplementary Fig. 3a. The magnetoresistance is significant with abrupt changes around ±1 and ±2 T, consistent with the RMCD result (Fig. 4b). The *I*_dark_−*V* curves at three representative magnetic field (0, 1.5, and 3 T) are shown in Supplementary Fig. 3b. We can see that, for the applied magnetic fields in our experiment, the tunneling currents in dark are negligible within *V* = ±0.19 V. To eliminate the influence of the tunneling current (to be discussed in the next section), herein, the photocurrents are measured at *V* = −0.15 V under various magnetic fields by the lock-in technique. Both the RMCD signal (Fig. 4b) and photoresponsivity polarization $\rho$ (Fig. 4c) show five plateaus when *μ*_0_*H* sweeps between −3.5 and +3.5 T, corresponding to five magnetic states enabled by the layered AFM nature of multilayer CrI_3_. The photoresponsivity polarization $\rho$ equals 0 at *μ*_0_*H* = 0 T, corresponding to the AFM ground state, and saturates at ±4.5% when | *μ*_0_*H* | > 2.2 T, corresponding to the fully spin-polarized states.

### The abnormal negative photocurrents at higher bias

**Fig. 5:**
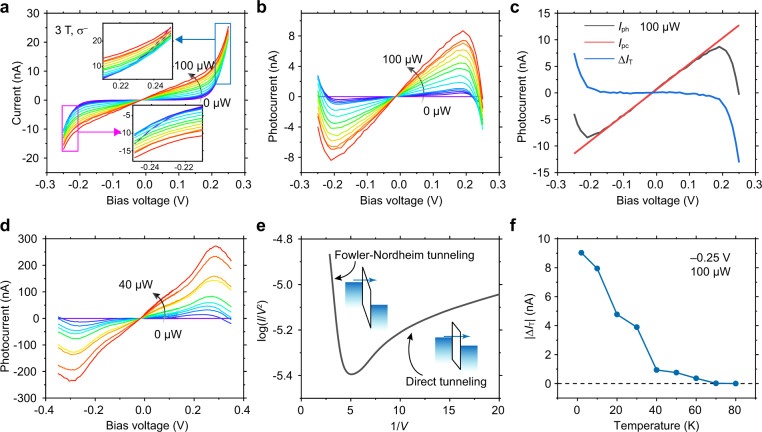
The abnormal negative photocurrent in the CrI_3_ devices. **a** *I*–*V* curves of the ~10 nm thick CrI_3_ light helicity detector D3 under ${\sigma }^{-}$ excitation with various powers (from 0 to 100 μW) at *μ*_0_*H* = 3 T. The insets: the zoomed-in regions at higher bias. The *I*_light_–*V* curves are linear within *V* = ±0.19 V, but become nonlinear and cross to each other at higher bias. **b** The photocurrents *I*_ph_ obtained from (**a**) by subtracting *I*_dark_ from *I*_light_. For all the excitation powers, the *I*_ph_ increases linearly with bias within ±0.19 V, but deviates from linear behavior and even decreases at higher bias, resulting in a negative photocurrent. **c** The *I*_ph_–*V* curve under 100 μW excitation (black line), the *I*_pc_–*V* curve extracted by linear fitting the *I*_ph_–*V* curve within *V* = ±0.15 V, and extending the result to the full bias range of ±0.25 V (red line), and the Δ*I*_T_–*V* curve (blue line) obtained by subtracting *I*_pc_ from *I*_ph_. Under light illumination, the tunneling current decreases for bias voltages |*V* | > 0.19 V. **d** The *I*_ph_–*V* curves in the monolayer CrI_3_ device D1 under various excitation powers at *μ*_0_*H* = 0 T. The negative photocurrent phenomenon occurs at bias higher than 0.25 V. **e** The dark tunneling currents in the monolayer CrI_3_ device D1 are plotted in the log(*I*/*V*^2^)–1/*V* diagram. The negative (positive) slope of the curve at high (low) bias region indicates the Fowler–Nordheim tunneling (direct tunneling) mechanism. The two insets: the energy band diagrams of the two tunneling mechanism, the left for the Fowler–Nordheim tunneling and the right for the direct tunneling. **f** The temperature-dependent | Δ*I*_T_ | in D3 measured at *V* = −0.25 V under 100 μW light excitation. With the temperature increasing, |Δ*I*_T_ | decreases monotonously until zero (the dashed line) at a temperature higher than 60 K.

The *I*–*V* curves of a ~10 nm thick CrI_3_ light helicity detector D3 are measured at *μ*_0_*H* = 3 T under ${\sigma }^{-}$ excitation with various powers (from 0 to 100 μW) (Fig. 5a). The *I*–*V* curves are linear within *V* = ±0.19 V, but become nonlinear and cross to each other at higher biases (e.g., 0.25 V, the insets in Fig. 5a). Specifically, for identical bias within ±0.19 V, the *I*_light_ increases monotonically with the excitation power increasing. However, it decreases first and then increases with the excitation power increasing at a higher bias, e.g., *V* = 0.25 V (Supplementary Fig. 4). Such intriguing phenomenon can be reproduced in D2 (Supplementary Fig. 5). We extract the photocurrents *I*_ph_ by subtracting *I*_dark_ from *I*_light_ (Fig. 5a) as shown in Fig. 5b. We can see that, for all the excitation powers, *I*_ph_ increases linearly with bias within *V* = ±0.19 V, but deviates from linear behavior and even decreases at higher bias, resulting in a negative photocurrent. The critical biases of ±0.19 V coincide with the bias at which *I*_dark_ becomes significant (see the 0 μW excited *I*–*V* curve in Fig. 5a).

In order to explore the abnormal negative photocurrent phenomenon in multilayer CrI_3_ devices, we consider that the *I*_light_ includes the photocurrent *I*_ph_ and the tunneling current in dark *I*_dark_. The *I*_ph_ includes the photoconductive current *I*_pc_ and the variation of the tunneling current under light illumination Δ*I*_T_. Given a linear relationship between the *I*_pc_ and applied bias *V*, we extract the photoconductivity current *I*_pc_ by linear fitting the *I*_ph_–*V* curve (under 100 μW excitation) within *V* = ±0.15 V, and extending the result to the full bias range of ±0.25 V (red line in Fig. 5c). Next, we obtain the Δ*I*_T_ (blue line in Fig. 5c) by subtracting *I*_pc_ (red line) from the photocurrent *I*_ph_ (black line). We can see that, under light illumination, the tunneling current reduces at a higher bias (|*V* | > 0.19 V), and the reduction magnitude | Δ*I*_T_ | increases monotonically with excitation power (Supplementary Fig. 6). If normalize the Δ*I*_T_–*V* and *I*_dark_–*V* curves by their respective current values at *V* = 0.25 V, we find that the two curves overlap well with each other (Supplementary Fig. 7), indicating that the Δ*I*_T_ is proportional to the *I*_dark_, regardless of the applied bias. These results suggest that the abnormal negative photocurrent phenomenon at higher bias in multilayer CrI_3_ devices may result from tunneling current reduction under light illumination.

It is worth noting that the negative photocurrent phenomenon can also be observed in the monolayer CrI_3_ device at a higher bias (|*V* | > 0.25 V, Fig. 5d). We plot the dark tunneling current in a log(*I*/*V*^2^) vs. 1/*V* diagram^15,16^ to examine the tunneling mechanisms in both monolayer and multilayer CrI_3_. The log(*I*/*V*^2^)−1/*V* curve for monolayer device (Fig. 5e) shows negative (positive) slope at higher (lower) bias region, corresponding to Fowler–Nordheim (FN) tunneling (direct tunneling)^17^. Direct tunneling occurs in the lower bias region, i.e., the carriers directly tunnel through a trapezoidal barrier (the right inset in Fig. 5e) without entering into the conduction band of semiconductor^18^. FN tunneling occurs in a higher bias region (>0.25 V for monolayer CrI_3_ case), wherein the energy band bends more and the electrons tunnel into the conduction band of the semiconductor (the left inset in Fig. 5e)^6,8^. The log(*I*/*V*2)−1/*V* curve for the ~10-nm CrI_3_ device D3 is shown in Supplementary Fig. 8, where the FN tunneling occurs at *V* > 0.19 V. These results indicate that there exists a relationship between the abnormal photocurrent and the tunneling current with FN tunneling mechanism.

We further perform the temperature-dependent measurement in the ~10 nm thick CrI_3_ device D3. As temperature increases, the | Δ*I*_T_ | (measured at *V* = −0.25 V) exhibits a monotonous decrease until zero at a temperature higher than the Curie temperature of CrI_3_ (*T*_c_ = 61 K^4^). This result indicates that the negative photocurrent phenomenon weakens as temperature increases, and vanishes at temperature higher than 60 K. The temperature dependence of the dark tunneling current *I*_dark_ for the 10-nm CrI_3_ device is shown in Supplementary Fig. 9a. As the temperature increases from 2 K, the *I*_dark_ first decreases and then increases. The turning point is 60 K. Similar result was reported previously for CrI_3_ tunneling device^8^. The *I*_ph_−*V* relations at various temperatures are shown in Supplementary Fig. 9b. At lower temperature, the *I*_ph_−*V* curves are nonlinear due to the negative photocurrent. As temperature increases, the *I*_ph_−*V* curves become linear, corresponding to the weakening negative photocurrent phenomenon.

One possible origin for the abnormal negative photocurrent phenomenon is the light-induced defects in CrI_3_^19^. The defects may trap the electrons which tunnel into the conduction band of CrI_3_ through FN tunneling, resulting in reduced conductivity. At higher temperatures (*k*_B_*T* > activation energy of the trapped electron), the trapped electrons would release and contribute again to conductivity. Therefore, the absolute value of Δ*I*_T_ decreases with increasing temperature as shown in Fig. 5f. Nevertheless, to fully verify this, further study of dynamic process of the interaction between photons and electrons is needed.

## Discussion

In conclusion, we fabricated light helicity detectors based on graphene-CrI_3_-graphene vdW heterostructures and measured their circularly polarized light excited current and RMCD under various magnetic fields. The helicity-dependent photocurrent has a clear relation with a magnetic field, consistent with the RMCD result. For the monolayer CrI_3_ device D1, the photoresponsivity polarization switches between ±6% as the magnetic field sweeps between ±0.3 T. The helicity-dependent photoresponse phenomenon vanishes at temperature higher than 40 K, close to the Curie temperature (*T*_c_) of monolayer CrI_3_ (45 K). For the multilayer CrI_3_ device, the photoresponsivity polarization performs AFM properties that saturates at ±4.5% when | *μ*_0_*H* | > 2.2 T, and equals 0 at *μ*_0_*H* = 0 T. Moreover, we find abnormal negative photocurrent phenomenon at higher bias in both monolayer and multilayer CrI_3_. We try to unveil the origin of this phenomenon by investigating the tunneling current mechanism in CrI_3_, together with the temperature dependent measurement. Our work reveals the interplay between magnetic and optoelectronic properties in CrI_3_ and paves the way to developing spin-optoelectronic devices.

## Methods

### Device fabrication

First, few-layer graphene, CrI_3_, and *h*BN (10–30 nm) flakes were exfoliated onto polydimethylsiloxane (PDMS) substrates in an argon gas-filled glove box with <0.1 ppm concentration of oxygen and water. Then, the *h*BN encapsulated graphene-CrI_3_-graphene heterostructure was assembled layer-by-layer on a Si/SiO_2_ substrate in the same glove box with the dry transferring method using the PDMS substrate as stamp under the help of a microscope attached with a micro-manipulator. The two graphene electrodes were contacted to the pre-prepared Cr/Au (5/25 nm) electrodes for measuring purpose. The thicknesses of the flakes were estimated by optical contrast and confirmed by atomic force microscopy after device measurements.

### RMCD and magneto-optoelectronic measurements

Both RMCD and magneto-optoelectronic measurements were performed in a dry cryostat (attoDRY2100, base temperature of 1.7 K) equipped with a 9 T superconducting magnet and a home tailored microscopic setup in back-scattering geometry. The RMCD is defined as $({R}_{\sigma +}-{R}_{\sigma -})/({R}_{\sigma +}+{R}_{\sigma -})$, where ${R}_{\sigma \pm }$ are the intensities of the ${\sigma }_{\pm }$ reflected light. For RMCD measurement, the temperature was 2 K, and the device was illuminated by a 633 nm He-Ne laser with a power of 10 μW. The laser was modulated by a chopper and a photoelastic modulator (PEM100), and was focused on the device with a beam diameter ~1 μm by a low-temperature apochromatic objective (LT-APO/VISIR/0.82). The collected light from the sample via the same objective was sent to a photomultiplier tube (PMT1001) equipped with a lock-in amplifier (HF2LI). For magneto-optoelectronic measurement, circularly polarized light was realized by modulating the 633 nm He-Ne laser with a polarizer and a quarter wave plate equipped with a motorized rotation stage. A chopper was employed to switch the light between on and off states with a frequency of 73 Hz. The photocurrent was measured directly by the lock-in amplifier. The *I*–*V* curves were measured with a Keithley 2636B source/measure unit. The temperature was 2 K and the excitation power was 10 μW, unless otherwise specified.

## Supplementary information

Supplementary Information

## Data Availability

All relevant data are available from the corresponding author on request.
