# Supplementary material for: Light helicity detector based on 2D magnetic semiconductor CrI3
Source: Nat Commun. 2021 Nov 25;12:6874. doi: 10.1038/s41467-021-27218-3 (PMC8617301; doi:10.1038/s41467-021-27218-3)
Supplement: Supplementary file 1 — Supplementary Information [file 41467_2021_27218_MOESM1_ESM.pdf]

Supplementary Information for

Light helicity detector based on 2D magnetic semiconductor CrI_3

Xing Cheng, Zhixuan Cheng, Cong Wang, Minglai Li, Pingfan Gu, Shiqi Yang, Yanping Li, Kenji Watanabe, Takashi Taniguchi, Wei Ji, Lun Dai*

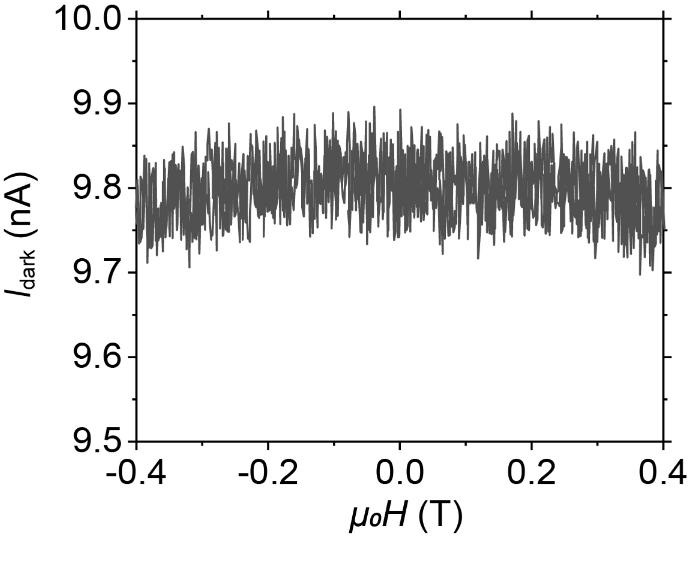

Supplementary Figure 1 | The dark tunneling current I_{dark} in the monolayer CrI_3. The I_{dark} is almost independent of the magnetic field, because the resistances of the down- or up-magnetized monolayer CrI_3 are equal.

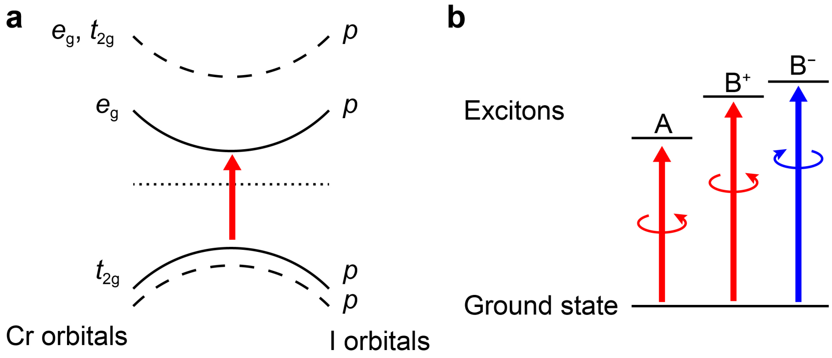

Supplementary Figure 2 | Optical transitions in CrI_3 under the 633 nm light excitation. a Schematic diagram of the splitting band structure in a fully magnetized CrI_3. The solid (dashed) lines denote the majority-spin (minority-spin) polarization. The dotted line denotes the Fermi level. The gaps are different for majority and minority spins. **b** Three bright exciton states dominated by transitions between the majority-spin valence and conduction bands of ferromagnetic monolayer CrI_3. Under the 633 nm excitation, only excitons related to transition between majority spin valence and conduction bands are created, which obeys the light helicity-selective transition rule. As a result, the absorption is different for σ^+ and σ^- light. For more detailed discussion, please refer to Fig. 3j in *Nat. Commun.* **10**, 2371 (2019), and *Nat. Phys.* **14**, 277 (2018).

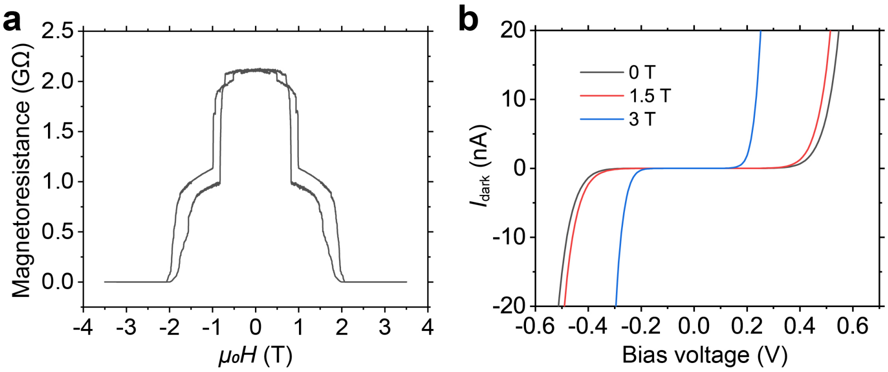

Supplementary Figure 3 | The dark tunneling currents in D2. a The corresponding magnetoresistance under various magnetic field measured at $V = 0.3$ V, $T = 2$ K. The magnetoresistance is significant with abrupt changes around ±1 T and ±2 T, consistent with the RMCD result. **b** The $I_{dark}-V$ curves at three representative magnetic field (0, 1.5, and 3 T). For the applied magnetic fields in our experiment, the dark tunneling currents are negligible within $V = \pm 0.15$ V.

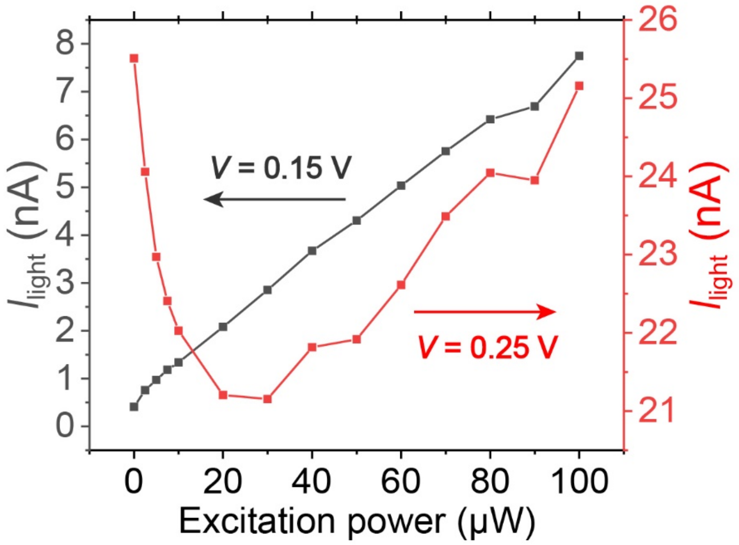

Supplementary Figure 4 | Excitation power dependence of light-on currents. Excitation power dependence of light-on currents of the ~10 nm thick CrI_3 light helicity detector D3 under μ_0H = 3 T measured at V = 0.15 and 0.25 V. The I_{light} increases monotonically with excitation power at lower bias of 0.15 V, but decreases first and then increases with excitation power at higher bias of 0.25 V.

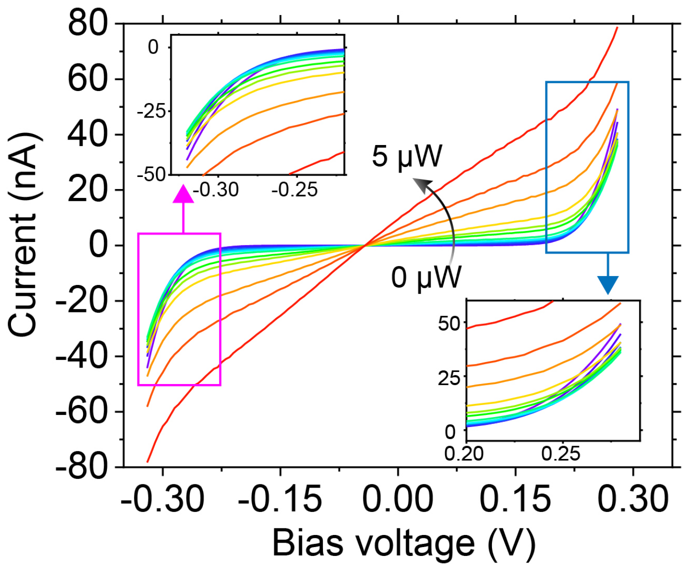

Supplementary Figure 5 | Excitation power dependent I_{light}–V curves of D2. I–V curves of the 14 nm thick CrI_3 light helicity detector D2 under σ^- excitation with various powers (from 0 to 5 μW) at μ_0H = 3 T. The insets: the zoomed-in regions at higher biases. The I_{light}–V curves are linear within ±0.15 V, but become non-linear and cross to each other at higher biases.

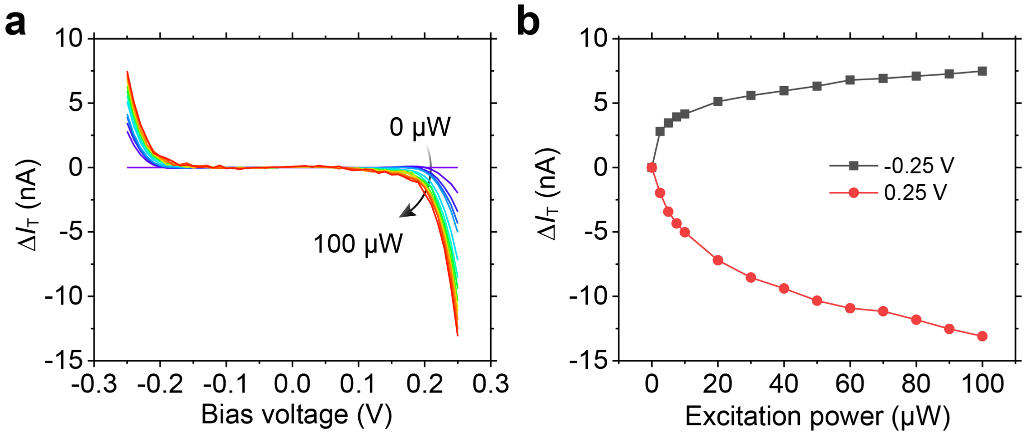

Supplementary Figure 6 | The excitation power dependent ΔI_T of D3. a The ΔI_T–V curves measured under various excitation powers. **b** The excitation power dependent ΔI_T at fixed biases of −0.25 V and +0.25 V. At higher biases as ±0.25 V, the reduction magnitude $|\Delta I_T|$ increases monotonically with excitation power.

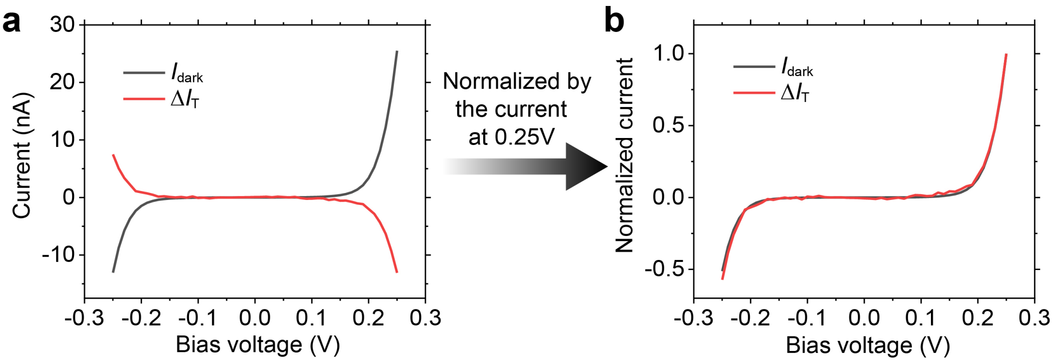

Supplementary Figure 7 | Comparison of I_{dark} and ΔI_T of D3. a The I_{dark}–V (black) curve obtained from Fig. 5a and the ΔI_T–V (red) curve obtained from Fig. 5c in the manuscript. **b** Normalized curves obtained from **a** by their respective current values at $V = 0.25$ V. The two curves overlap well with each other, indicating that the tunneling current reduces proportionally under light illumination.

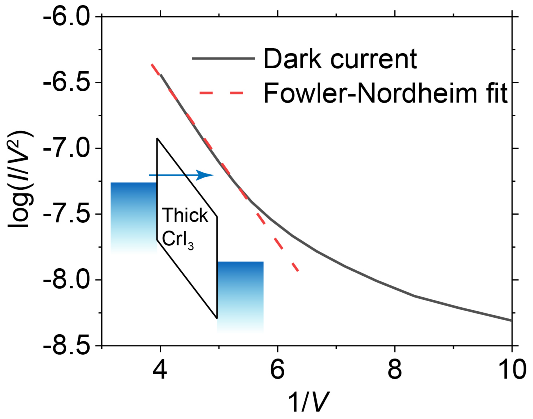

Supplementary Figure 8 | The dark tunneling currents in D3. The dark tunneling currents in the ~10-nm CrI_3 device D3 plotted in the $\log(I/V^2)$–$1/V$ diagram. The dashed red line is the Fowler-Nordheim tunneling fitting result. The inset: the energy band diagram of a thick CrI_3 with triangular barrier, in which Fowler-Nordheim tunneling occurs.

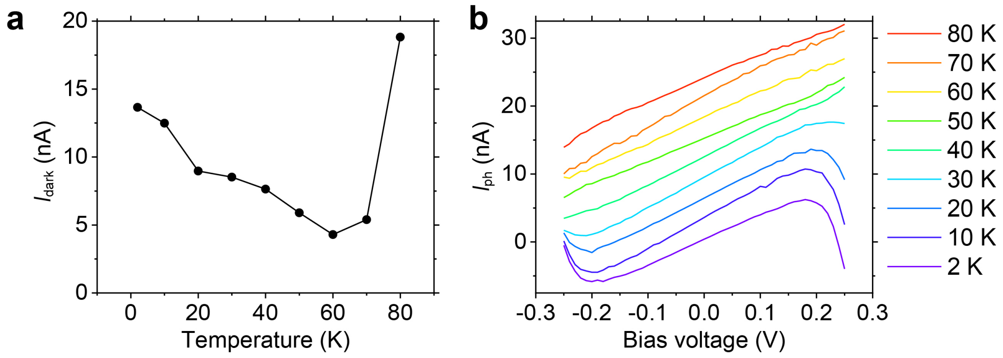

Supplementary Figure 9 | Temperature dependent measurements for D3. a The temperature dependence of the dark tunneling current I_{dark}. As temperature increases from 2 K, the I_{dark} decreases first until 60 K, and then increases. **b** The I_{ph}–V curves at various temperatures, from 2 to 80 K. At lower temperature, the I_{ph}–V curves are nonlinear because of the negative photocurrent. As temperature increases, the negative photocurrent phenomenon weakens, and eventually vanishes at temperature higher than 60 K. Meanwhile, the I_{ph}–V curves become linear at higher temperature.
